# Deregulation of HDAC5 by Viral Interferon Regulatory Factor 3 Plays an Essential Role in Kaposi's Sarcoma-Associated Herpesvirus-Induced Lymphangiogenesis

**DOI:** 10.1128/mBio.02217-17

**Published:** 2018-01-16

**Authors:** Hye-Ra Lee, Fan Li, Un Yung Choi, Hye Ryun Yu, Grace M. Aldrovandi, Pinghui Feng, Shou-Jiang Gao, Young-Kwon Hong, Jae U. Jung

**Affiliations:** a Department of Biotechnology and Bioinformatics, College of Science and Technology, Korea University, Sejong, South Korea; b Department of Pediatrics, Children’s Hospital Los Angeles, Los Angeles, California, USA; c Department of Pediatrics, UCLA Medical School, Los Angeles, California, USA; d Department of Molecular Microbiology and Immunology, Keck School of Medicine, University of Southern California, Los Angeles, Los Angeles, California, USA; e Department of Surgery, Keck School of Medicine, University of Southern California, Los Angeles, Los Angeles, California, USA; Icahn School of Medicine at Mount Sinai

**Keywords:** Kaposi's sarcoma-associated herpesvirus, angiogenesis, histone deacetylase

## Abstract

Kaposi’s sarcoma-associated herpesvirus (KSHV) is the etiologic agent for Kaposi’s sarcoma (KS), which is one of the most common HIV-associated neoplasms. The endothelium is the thin layer of squamous cells where vascular blood endothelial cells (BECs) line the interior surface of blood vessels and lymphatic endothelial cells (LECs) are in direct contact with lymphatic vessels. The KS lesions contain a prominent compartment of neoplastic spindle morphology cells that are closely related to LECs. Furthermore, while KSHV can infect both LECs and BECs *in vitro*, its infection activates genetic programming related to lymphatic endothelial cell fate, suggesting that lymphangiogenic pathways are involved in KSHV infection and malignancy. Here, we report for the first time that viral interferon regulatory factor 3 (vIRF3) is readily detected in over 40% of KS lesions and that vIRF3 functions as a proangiogenic factor, inducing hypersprouting formation and abnormal growth in a LEC-specific manner. Mass spectrometry analysis revealed that vIRF3 interacted with histone deacetylase 5 (HDAC5), which is a signal-responsive regulator for vascular homeostasis. This interaction blocked the phosphorylation-dependent cytosolic translocation of HDAC5 and ultimately altered global gene expression in LECs but not in BECs. Consequently, vIRF3 robustly induced spindle morphology and hypersprouting formation of LECs but not BECs. Finally, KSHV infection led to the hypersprouting formation of LECs, whereas infection with a ΔvIRF3 mutant did not do so. Collectively, our data indicate that vIRF3 alters global gene expression and induces a hypersprouting formation in an HDAC5-binding-dependent and LEC-specific manner, ultimately contributing to KSHV-associated pathogenesis.

## IMPORTANCE

Several lines of evidences indicate that KSHV infection of LECs induces pathological lymphangiogenesis and that the results resemble KS-like spindle morphology. However, the underlying molecular mechanism remains unclear. Here, we demonstrated that KSHV vIRF3 is readily detected in over 40% of various KS lesions and functions as a potent prolymphangiogenic factor by blocking the phosphorylation-dependent cytosolic translocation of HDAC5, which in turn modulates global gene expression in LECs. Consequently, vIRF3-HDAC5 interaction contributes to virus-induced lymphangiogenesis. The results of this study suggest that KSHV vIRF3 plays a crucial role in KSHV-induced malignancy.

## INTRODUCTION

Kaposi’s sarcoma-associated herpesvirus (KSHV) has been identified as an etiologic agent of multicentric Castleman’s disease (MCD), primary effusion lymphoma (PEL), and Kaposi’s sarcoma (KS) (1–3). KS is a neoplasia of lymphatic endothelial origins that is characterized by a prominent compartment of spindle cells (SCs), which harbor mainly latent KSHV (4, 5). Furthermore, previous studies showed that KSHV infection activates genetic programming related to lymphatic endothelial cell (LEC) fate, suggesting that lymphangiogenic pathways are involved in KSHV infection and malignancy (6–8). Mounting data show that overexpression of several KSHV genes directly induces a number of angiogenic and/or lymphangiogenic pathways (7, 9). Even though accumulated studies suggest that LECs rather than vascular blood endothelial cells (BECs) are the primary precursors of KS, the mechanism by which KSHV induces lymphatic growth pathways remains unclear.

Angiogenesis, the process of formation of new blood vessels from preexisting ones, is a physiological and pathological process that initiates from quiescent endothelium being activated by proangiogenic stimuli (10, 11). Once activated, endothelial cells migrate and form sprouts that are involved in characteristic changes of the gene expression profile. Recently, histone deacetylase 5 (HDAC5), a member of the class IIa HDACs, has emerged as a crucial signal-responsive regulator of gene expression involved in vascular homeostasis (12–15). Remarkably, unlike other HDAC families, class IIa HDACs lack measurable enzymatic activity (14). Thus, in order to regulate expression of their target genes, members of the class IIa HDACs utilize signaling-dependent nucleocytoplasmic shuttling (15, 16). For instance, upon stress, HDAC5 can be phosphorylated and translocated to the cytoplasm; this ultimately leads to anti-angiogenic gene expression, suggesting that HDAC5 is a crucial proangiogenic factor in the endothelium (12, 17).

The KSHV genome encodes four types of viral interferon regulatory factors (vIRFs), vIRF1 to vIRF4, which are homologous to cellular IRFs, which are clustered within one genomic locus (18–20). Although all vIRFs inhibit both host interferon response and control cellular growth, differences in their activation characteristics result in distinct immune evasion strategies. This enables efficient lifelong persistence and facilitates the development of KSHV-associated malignancy. Expression of vIRF3 is required for continuous proliferation of PEL cells (21) and causes dramatic changes in critical host pathways such as apoptosis, cell cycle, antiviral immune response, and tumorigenesis (9, 18–20). It was previously shown that vIRF3 (also referred to as LANA-2) is constitutively expressed in B cell lymphotropic diseases, PEL, and MCD but not in KS tumors (22). Taken together, these findings indicate that vIRF3 is a crucial factor for maintenance of KSHV-associated lymphoproliferative disease. Nevertheless, the biological function of vIRF3 in KS is not clear.

In the present study, we found that KSHV vIRF3 was readily detected in over 40% of KS lesions and that it interacted directly with HDAC5, leading to the decreased phosphorylation of HDAC5 and retention of HDAC5 activity in the nucleus. Consequently, vIRF3 expression altered global gene expression of LECs and facilitated hypersprouting formation in LECs. Collectively, our results demonstrate the crucial role of vIRF3 as a driving force in KSHV-associated pathogenesis.

## RESULTS

### KSHV vIRF3 is expressed in KS lesions.

**FIG 1:**
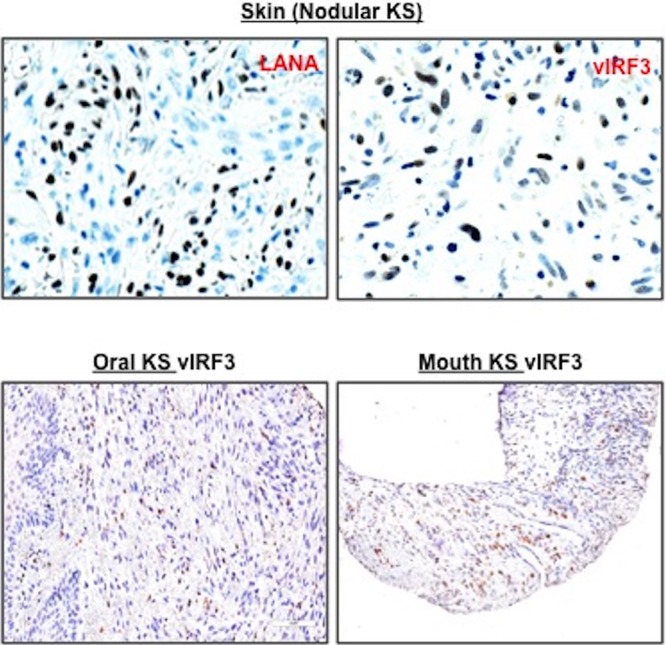
vIRF3 expression in KS tissues. We obtained KS tissue microarrays from AIDS Cancer Specimen Resource and performed immunohistochemistry, visualized by Aperio F.L. digital pathological scanning. A biopsy sample of KS TMA was stained with anti-LANA and anti-vIRF3 (CM-A807). Representative images show the LANA staining of skin KS lesion and the vIRF3 staining of skin, tonsil, and mouth KS lesions and positive staining corresponding to either LANA or vIRF3 in total KS biopsy specimens embedded in TMA.

Among the vIRFs, vIRF3 is latently expressed and required for the proliferation of virus-induced B cell lymphoma (18, 21, 22). However, it is unclear whether vIRF3 is expressed in KS lesions. To elucidate the expression of vIRF3 in KS, we performed tissue microarray (TMA) analysis of KS tissue samples, consisting of 11 normal biopsy specimens and 50 KS biopsy specimens from various organs, by immunohistochemistry using a CM-A807 mouse monoclonal antibody against vIRF3. In contrast to a previous report that vIRF3 is exclusively expressed in B cell lymphoproliferative diseases, our results showed that vIRF3 was expressed in ~42% of KS tissues from various organs, including skin, lymph node, mouth, head/neck, tonsil, and rectum KS samples (Fig. 1 and Table 1). In 11 normal tissue specimens, no vIRF3 staining was observed. Results obtained using the LANA antibody for KS TMA corroborated the results obtained using CM-A807 monoclonal antibody (Table 1). Interestingly, consistent with the nuclear-cytoplasmic shuttling property of vIRF3 (23), 7 of the 21 vIRF3-positive KS specimens showed both nuclear and cytoplasmic staining, while 14 of the 21 vIRF3-positive specimens were mainly enriched in the nucleus (Fig. 1 and Table 1). This observation shows that vIRF3 is expressed in ~42% of KS tissues from various organs.

**TABLE 1:** Immunohistochemistry of vIRF3 expression in KS tissue lesions^a^

Tissue type and biopsy specimen ID	LANA (1:500)	vIRF3 (1:750)	Organ
Normal			
1	No	No	Lymph node
2		No	Lymph node
4	No	No	Lymph node
31		No	Lymph node
42	No		Lymph node
55	No	No	Lymph node
91	No	No	Lymph node
92	No	No	Lymph node
93	No	No	Lymph node
94	No	No	Lymph node
99		No	Lymph node
KS			
8	Positive	Positive	Skin
9	Positive	Positive+	Skin
15	Positive	Positive+	Skin
16		Positive+	
18	Positive	Positive	Skin
19		No	Skin
20	Positive	Positive+	Skin
24	No	Positive+	Mouth
25	No	No	Mouth
27	No	No	Lymph node
28	No	Positive+	Lymph node
29		No	Lymph node
30	No		Lymph node
37	Positive	No	Skin
38		No	Skin
58	Positive		Skin
62	No	Positive	Head/neck
65	Positive	No	Tonsil
66	Positive		Tonsil
67	Positive	No	Head/neck
73	Positive		Lymph node
74	Positive		Lymph node
87	Positive	Positive	Skin
88	Positive	Positive	Skin
89	Positive	Positive	Mouth
90	Positive	Positive	Mouth
95	Positive	Positive	Mouth
97	No	No	Lymph node
109	No		Tonsil
119	No	No	Skin
121	No	Positive	Skin
128	No	No	Skin
136	No	Positive	Lymph node
147	No	No	Mouth
153/167	Positive	Positive	Mouth (skin)
157	Positive	Positive+	Lymph node
161	Positive	No	Mouth
162	Positive	No	Mouth
171	Positive	Positive	Mouth
172	Positive	Positive	Tonsil
174	No	No	Skin
183		No	Skin
187	No		Mouth
190	No	Positive	Rectum
195	Positive	No	Skin
196	Positive	No	Skin
198	Positive	No	Skin
200	Positive	No	Anus
203	No	No	Skin
215	No	No	Lymph node

a Blank cells indicate that no tissue was embedded; "Positive" indicates nuclear staining of vIRF3; "Positive+" indicates nuclear and cytoplasmic staining of vIRF3. Zero of 11 (0%) and zero of 11 (0%) normal tissue specimens gave positive or positive+ results for LANA and vIRF3, respectively; 27 of 50 (54%) and 21 of 50 (42%) KS specimens gave positive or positive+ results for LANA and vIRF3, respectively. ID, identifier.

### KSHV vIRF3 induces hypersprouting formation in lymphatic endothelial cells but not in blood endothelial cells.

**FIG 2:**
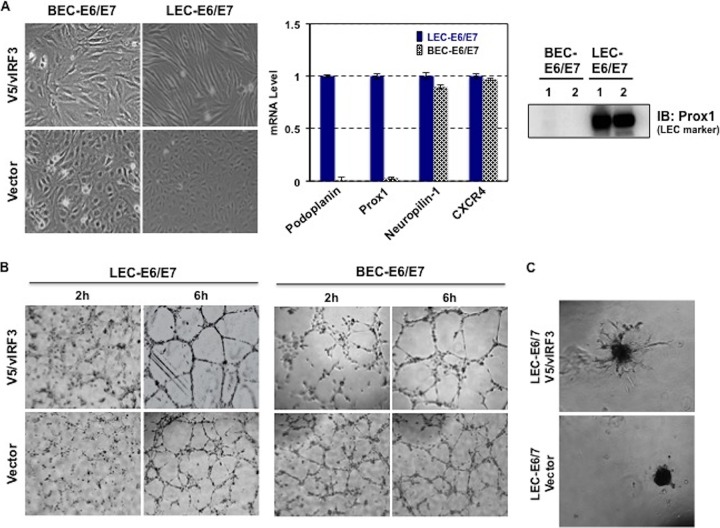
vIRF3 expression facilitates sprouting formation in a LEC-specific manner. (A) Induction of spindle morphogenesis by vIRF3 in LECs but not in BECs (left panel) and real-time RT-PCR measurement of levels of podoplanin, Prox1, Neutropilin-1, and CXCR4 from LEC-E6/E7 and BEC-E6/E7 cells (middle panel). Prox1 expression was detected in LEC-E6/E7 cells (right panel). IB, immunoblot. (B) Tube formation assay. Either BECs expressing vector and vIRF3 or LECs expressing vector and vIRF3 were tested. The assay was monitored at the indicated time points. (C) Sprouting formation assay. Assays were performed on matrigel with LEC-E6/E7 vector and LEC-E6/E7 V5/vIRF3.

In order to understand the biological function of vIRF3 in highly angiogenic KS, we first generated E6/E7 immortalized LEC and BEC cell lines that constitutively expressed vIRF3. To further validate the E6/E7 protein effects on LECs and BECs, we choose representative unique genes of LECs and BECs and performed real-time PCR (RT-PCR) along with immunoblotting. Notably, without any change in the characteristics of LECs and BECs, as ascertained by RT-PCR of either LEC- or BEC-specific markers, vIRF3 induced elongated spindle cell morphology in LECs but not in BECs (Fig. 2A). Prox1 and podoplanin were used as positive markers for LECs (24) and CXCR4 and neuropilin-1 were employed as positive markers for BECs (25). To examine whether vIRF3 has the ability to induce angiogenesis, we performed capillary-like tube formation assays using BEC-E6/E7-vector, BEC-E6/E7-V5/vIRF3, LEC-E6/E7-vector, and LEC-E6/E7-V5/vIRF3. We found that at 6 h after seeding the cells onto matrigel matrix, both BEC-E6/E7-V5/vIRF3 and LEC-E6/E7-V5/vIRF3 readily formed tubes compared with BEC-E6/E7-vector and LEC-E6/E7-vector (Fig. 2B). We also prepared cell spheroid assays from BEC-E6/E7-vector, BEC-E6/E7-V5/vIRF3, LEC-E6/E7-vector, and LEC-E6/E7-V5/vIRF3. vIRF3 actively induced sprouting formation in LECs (Fig. 2C) but not in BECs (data not shown). These data indicate that KSHV vIRF3 expression leads to lymphatic endothelial capillary sprouting.

### vIRF3 is required for KSHV-mediated transformation of LECs.

**FIG 3:**
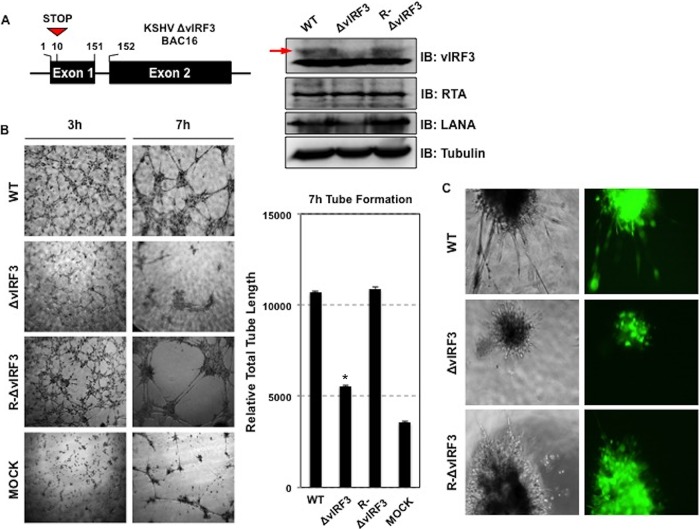
vIRF3 is required for KSHV-induced tube formation and sprouting formation. (A) (Left panel) Construction of KSHV ΔvIRF3 BAC16. A schematic diagram of KSHV ΔvIRF3 BAC16 construction is shown. (Right panel) Wild-type (WT), ΔvIRF3, and R-ΔvIRF3 recombinant KSHV-infected primary LECs were harvested at 72 h postinfection, and equal amounts of cell lysates were subjected to SDS-PAGE. (Right panel) Immunoblotting (IB) was performed with the indicated antibodies. (B and C) KSHV-induced tube formation and sprouting formation. (Left panel) WT, ΔvIRF3, and R-ΔvIRF3 recombinant KSHV-infected primary LECs were placed in Matrigel to perform sprouting formation and tube formation assays for the indicated times. (Right panel) Total tube lengths formed during the assay were measured by tube formation ACAS image analysis. *, *P* < 0.05. "MOCK" indicates KSHV-uninfected primary LECs. (C) Green fluorescence phase-contrast images of WT, ΔvIRF3, and R-ΔvIRF3 recombinant KSHV-infected primary LECs in matrigel are shown.

To examine the role of vIRF3 during lymphangiogenesis in the context of KSHV, we first generated a vIRF3 deletion mutant by adding a premature stop codon within *vIRF3* exon1 from BAC16 (KSHV-ΔvIRF3 BAC16) via “scarless” mutagenesis (26). To rule out the possibility of second-site mutations, we also constructed revertant clones in which the wild-type (WT) vIRF3 was restored (KSHV-R-ΔvIRF3 BAC16) (Fig. 3A). After verifying the recombinant constructs by DNA sequencing and enzyme digestion performed with NheI and AseI, we produced infectious virus according to a previously described method (26). We next determined whether deletion of vIRF3 had an effect on the production of infectious virus and viral gene expression. To this end, individual progeny viruses obtained from iSLK cells harboring KSHV-ΔvIRF3 BAC16, KSHV-R-ΔvIRF3 BAC16, and KSHV-BAC16 were used to infect primary LECs, and infection was quantified by green fluorescent protein (GFP) analysis. Levels of infection of primary LECs were comparable between WT and derivative viruses (see Fig. S1 in the supplemental material), suggesting that vIRF3 deletion does not affect virus production and infectivity. The lack of vIRF3 expression from KSHV-ΔvIRF3 BAC16 was confirmed (Fig. 3A, right panel). To further confirm that the vIRF3 gene is required for KSHV-mediated lymphatic endothelial cell sprouting, primary LECs were infected with WT, ΔvIRF3, and R-ΔvIRF3 BAC16 KSHV, followed by a tube formation assay performed on Matrigel. This showed that both WT and R-ΔvIRF3 KSHV induced capillary-like tube formation of primary LECs, whereas tube formation was dramatically reduced upon infection of recombinant ΔvIRF3 KSHV (Fig. 3B). Remarkably, both WT KSHV infection and R-ΔvIRF3 KSHV infection induced hypersprouting formation of primary LECs, whereas infection with ΔvIRF3 KSHV did not (Fig. 3C). Taken together, these results demonstrate that vIRF3 plays a critical role in KSHV-induced lymphatic endothelial cell sprouting.

10.1128/mBio.02217-17.1 FIG S1  Infectivity of recombinant KSHV in primary LECs. After the titer of each recombinant KSHV-BAC16 (KSHV-wt-BAC16, KSHV-ΔvIRF3 BAC16, and KSHV-R-ΔvIRF3 BAC16 from 293A) were determined by fluorescence microscopy and GFP-based flow cytometry, primary LECs were infected with similar titers of KSHV-BAC16, KSHV-ΔvIRF3 BAC16, or KSHV-R-ΔvIRF3 BAC16. Download FIG S1, JPG file, 2.9 MB. Copyright © 2018 Lee et al. 2018 Lee et al. This content is distributed under the terms of the Creative Commons Attribution 4.0 International license.

### vIRF3 reduces HDAC5 phosphorylation and thereby maintains the nuclear location of HDAC5.

**FIG 4:**
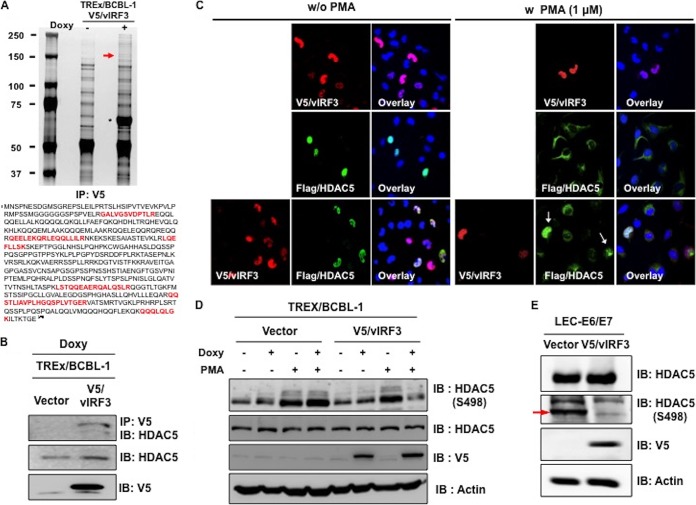
vIRF3-HDAC5 interaction decreases HDAC5 phosphorylation, blocking phosphorylation-dependent HDAC5 translocation. (A) Silver-stained purified V5-labeled vIRF3 complexes at 48 h after Doxy treatment of tetracycline-inducible V5-vIRF3-expressing cell lines. Asterisk, V5-vIRF3; arrow, HDAC5; IP, immunoprecipitation. (B) Interaction of vIRF3-HDAC5. Upon stimulation with Doxy (1 μg/ml), we performed IP with anti-V5, followed by IB with anti-HDAC5. (C) Effect of vIRF3 on the localization of HDAC5. HeLa cells were transiently transfected with the indicated constructs. Cells were then treated with PMA (1 μM) for 2 h before immunofluorescence staining was performed. Arrows indicate the cells coexpressing vIRF3 and HDAC5. (D) Effects of vIRF3 expression on the phosphorylation of HDAC5. At 24 h posttreatment with Doxy (1 μg/ml), TREx/BCBL-1 vector and TREx/BCBL-1 V5/vIRF3 cells were also treated with PMA (1 μM) for 2 h before being harvested. Equal amounts of total proteins were analyzed by IB using anti-HDAC5 pS498-specific antibody. (E) vIRF3 effect on HDAC5 phosphorylation in LECs. LEC-vector and LEC-V5/vIRF3 were harvested and IB was performed with anti-HDAC5 pS498-specific antibody along with other antibodies.

**FIG 5:**
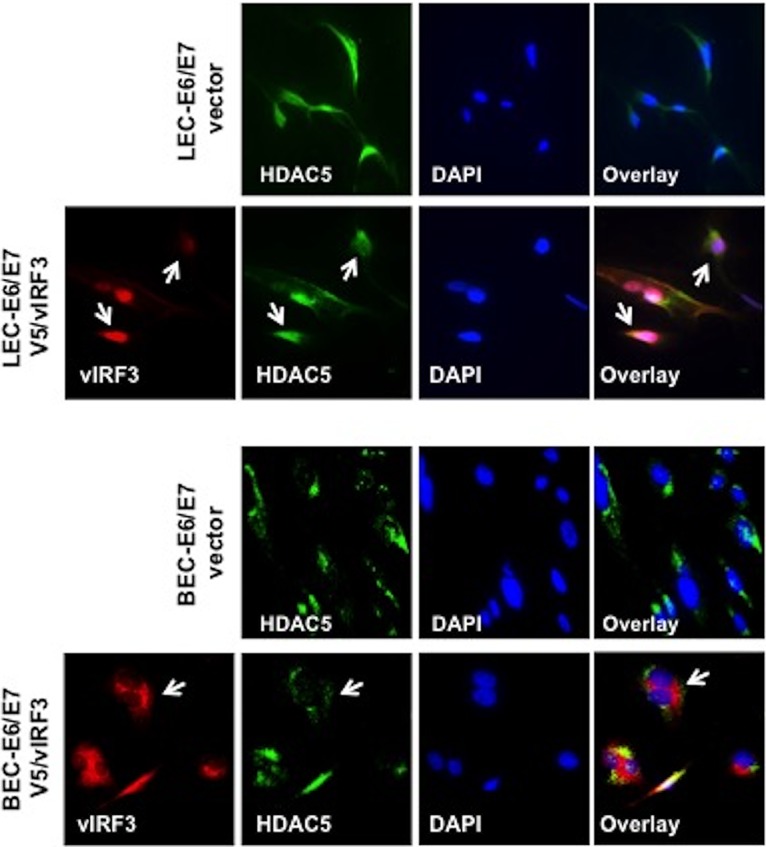
vIRF3 deregulates HDAC5 localization in LECs but not in BECs. Immunofluorescence staining for HDAC5 and vIRF3 was performed. BEC or LEC cells stably expressing vector or V5-vIRF3 were fixed and viewed by confocal microscopy using either anti-HDAC5 (green) or anti-V5 (red) antibodies. Nucleus (blue) was detected with Topro-3 staining.

To decipher the underlying molecular mechanism of vIRF3-induced hypersprouting formation in LECs, we performed mass spectrometry analysis to identify vIRF3-associated proteins using tetracycline-inducible V5-tagged vIRF3-expressing cell lines. TRExBCBL-1 V5/vIRF3 cells were treated with or without doxycycline (Doxy) for 24 h, followed by immunoprecipitation with an anti-V5 antibody. A polypeptide with a molecular mass of ~120 kDa was specifically found in Doxy-induced TRExBCBL-1 V5/vIRF3 cells. Mass spectrometry revealed this polypeptide to be histone deacetylase 5 (HDAC5) (Fig. 4A). We further confirmed that vIRF3 interacted with endogenous HDAC5 by immunoprecipitation (Fig. 4B). Previous studies reported that phorbol 12-myristate 13-acetate (PMA) phosphorylates HDAC5 at S259 and S498, leading to its nuclear export (27, 28). Having identified HDAC5 as a binding partner of vIRF3, we examined whether vIRF3 deregulated the nucleus-cytoplasm translocation of HDAC5 upon stimulation. To this end, cells were transfected with V5-vIRF3, Flag-HDAC5, or Flag-HDAC5 together with V5-vIRF3, followed by treatment with PMA to induce the phosphorylation of HDAC5. Confocal analysis showed that HDAC5 localized in the nucleus in the absence of treatment, while PMA treatment led to the nuclear export of HDAC5 (Fig. 4C). vIRF3 expression retained HDAC5 in the nucleus upon PMA treatment (Fig. 4C, arrows). Moreover, upon treatment of TREx/BCBL-1 vector and TREx/BCBL-1 V5/vIRF3 with Doxy and PMA, we observed a reduction of HDAC5 phosphorylation in vIRF3-expressing cells compared to vector-expressing cells (Fig. 4D). vIRF3 expression diminished a basal level of HDAC5 phosphorylation at serine 498 in LEC-V5/vIRF3 cells (Fig. 4E). We also found that HDAC5 was localized in both the nucleus and cytoplasm of LECs, while it was exclusively found in the cytoplasm of BECs. Coexpression of vIRF3 with HDAC5 resulted in the primarily nuclear localization of HDAC5 in LECs but not in BECs (Fig. 5). Collectively, these results show that vIRF3 expression significantly decreases the phosphorylation of HDAC5, retaining HDAC5 in the nucleus.

### HDAC5 is required for vIRF3-mediated hypersprouting formation in LECs.

**FIG 6:**
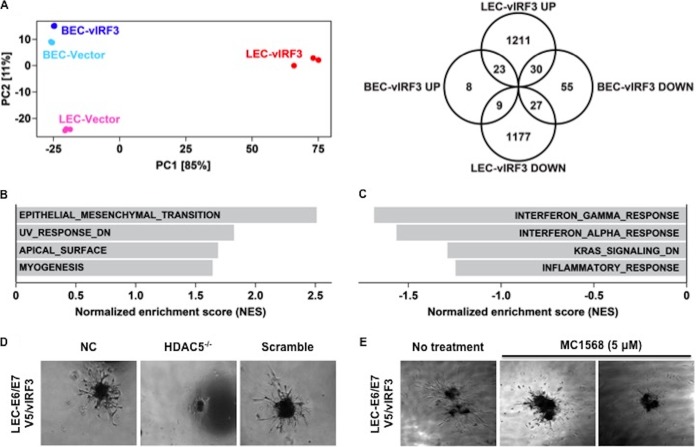
Alteration of the global gene expression of LECs by vIRF3 and the essential role of HDAC5 for vIRF3-mediated sprouting formation. (A) (Left panel) Principal component plot of both lymphatic endothelium gene expression and blood endothelium gene expression profiles upon vIRF3 expression. Numbers in brackets indicate percentages of total variation explained by each principal component (PC). (Right panel) Venn diagram showing the number of significantly up- and downregulated genes upon vIRF3 expression in LECs and BECs. (B and C) Gene set enrichment analysis (GSEA) of genes significantly up- or downregulated upon vIRF3 induction in LECs (B) and BECs (C), respectively. Positive normalized enrichment scores (NES) indicate increased pathway activity, and negative scores indicate decreased pathway activity. (D and E) The essential role of HDAC5 in vIRF3-induced sprouting formation. Phase-contrast images are shown. (D) LEC-E6/E7 V5/vIRF3 cells were transfected with siRNA as a control (Scramble) or with HDAC5-specific siRNA for 48 h, followed by embedding into matrigel. NC, negative control. (E) Sprouting of LEC-E6/E7 V5/vIRF3 spheroids treated with 5 μM MC1586 (HDAC5 inhibitor).

Because vIRF3 interaction deregulated HDAC5 nuclear-cytoplasmic shuttling, we investigated whether vIRF3 expression also affected HDAC5-mediated gene expression. RNA-Seq analysis revealed that, consistently with its LEC-specific activity for HDAC5 localization, vIRF3 expression induced large-scale changes in global gene expression only in LECs but not BECs (Fig. 6A, left panel). Using a cutoff false-discovery-rate (*q*) value of <0.05 and a fold change value of >4, we found 1,264 and 1,213 genes that were up- and downregulated by vIRF3 expression in LECs, respectively (Fig. 6A, right panel). In contrast, only 40 and 112 genes, respectively, were up- and downregulated by vIRF3 expression in BECs (Fig. 6A, right panel). Gene set enrichment analysis (GSEA) of these genes identified epithelial-cell-to-mesenchymal-cell transition, UV response, apical surface, and myogenesis as upregulated pathways in LECs (Fig. 6B; see also Table S1 in the supplemental material). Similar analysis in BECs revealed interferon gamma response, interferon alpha response, KRAS signaling, and inflammatory response as downregulated pathways in BECs (Fig. 6C and Table S1). Taken together, these results indicate noncanonical activities of vIRF3 in LECs, whereas vIRF3 expression in BECs appears to be mainly involved in IFN-mediated immune responses.

10.1128/mBio.02217-17.2 TABLE S1  Lists of gene set enrichment analysis data for genes significantly regulated upon vIRF3 induction in LECs and BECs. Download TABLE S1, DOCX file, 0.1 MB. Copyright © 2018 Lee et al. 2018 Lee et al. This content is distributed under the terms of the Creative Commons Attribution 4.0 International license.

Because vIRF3 interaction retained HDAC5 activity in the nucleus and led to altered expression of genes in LECs, we also investigated whether vIRF3-induced sprouting formation was dependent on HDAC5. Small interfering RNA (siRNA)-mediated knockdown of HDAC5 expression considerably suppressed sprout formation in LEC-V5/vIRF3 (Fig. 6D). Consistently with this observation, treatment with a class IIa HDAC inhibitor, MC1568, also robustly reduced sprouting formation of LEC-V5/vIRF3 (Fig. 6E). These results indicated that vIRF3-induced sprouting formation of LECs is dependent on HDAC5 expression and activity.

## DISCUSSION

The principal targets of KSHV infection in KS lesions are elongated spindle cells (SCs) thought to be of lymphatic endothelial origin (4). Thus, understanding the role of KSHV in the reprogramming of lymphatic endothelial cells is critical for understanding how KSHV infection causes KS tumors. However, it remains unclear which of the KSHV genes directly contribute to virus-induced reprogramming of LECs. Here, we demonstrate that KSHV vIRF3 is a prolymphangiogenic factor that dramatically alters the global gene expression of LECs via the deregulation of HDAC5 activity, ultimately inducing sprouting formation of LECs. Furthermore, we report for the first time that vIRF3 is detectable in approximately 42% of KS tissues from various organs, strongly suggesting a role of KSHV vIRF3 in KS pathogenesis.

Several studies have suggested that the original precursor of KS was of lymphatic endothelial lineage. First, KS seldom develops in organs lacking a lymphatic system (29–31). Second, LECs are more susceptible to KSHV infection than BECs (8). Third, KS-SCs mainly express LEC markers, as opposed to BEC markers, possibly through the induction of genetic reprogramming upon KSHV infection (6, 8). Recently, Cheng et al. showed that three-dimensional spheroid culture of KSHV-infected LECs leads to the induction of capillary sprouts and changes in gene expression profiles, such as endothelial-cell-to-mesenchymal-cell transition (EMT) markers (32). Consistently with these reports, evidence has indicated that vIRF3 directly contributes to the development of KS-SCs (1). vIRF3 was readily detected in various KS lesions (Fig. 1) (2). vIRF3-expressing LECs showed elongated spindle morphology, whereas BECs were not significantly altered by vIRF3 expression (Fig. 2A). Similar results were observed in KSHV-infected LECs but not in KSHV-infected BECs (3). vIRF3 expression induced hypersprouting formation in LECs (Fig. 2C). In line with this, KSHV infection led to hypersprouting formation of LECs, whereas infection with a ΔvIRF3 mutant did not do so (Fig. 3C) (4). Surprisingly, vIRF3 expression dramatically altered the transcriptome of LECs, particularly in EMT pathway-related genes (*q* < 0.05 and fold change > 4) (Fig. 6A and B). By contrast, vIRF3 expression in BECs mostly affected IFN pathway genes (Fig. 6C), suggesting that vIRF3 functions as an immune modulator in BECs.

Histone or protein deacetylases (HDACs) are enzymes with a critical role in diverse physiological and pathological processes. Mounting evidence shows that class IIa HDACs (HDAC4, HDAC5, HDAC6, HDAC7, and HDAC9) are essential factors for the angiogenic function of endothelial cells. Remarkably, the deacetylase activity of HDAC5 as well as of other class IIa HDACs is distinct from the conventional HDACs in that it occurs through signaling-dependent nucleocytoplasmic shuttling, which has an effect on the repression of anti-angiogenic genes in endothelium. Another peculiarity shared by the class IIa HDACs is that their N-terminal domain has highly conserved serine residues that undergo signal-dependent phosphorylation, ultimately leading to their nucleocytoplasmic shuttling. Our study shows that vIRF3 dramatically decreased HDAC5 phosphorylation, resulting in the retention of HDAC5 in the nucleus in LECs but not BECs (Fig. 4 and 5). At this point, the molecular mechanism by which vIRF3 interaction differentially regulates the phosphorylation of HDAC5 in LECs and BECs is not fully understood. It is reported that HDAC5 is phosphorylated by several different protein kinases (PKs), including protein kinase C (PKC), PKD, and Ca2^+^/calmodulin-dependent protein kinases (CaMKs) (13, 27, 33, 34). Interestingly, CaMKs lead to HDAC5 phosphorylation in skeletal muscle cells (33), while PKD induces the phosphorylation of HDAC5 in BECs (34). Therefore, one possibility is that other as-yet-unidentified kinases may be involved in the vIRF3-mediated reduction of HDAC5 phosphorylation in LECs. Additional studies will be necessary to determine the specific mode of action with regard to the vIRF3-HDAC5 interaction in LECs and BECs.

In summary, we have found that vIRF3 recapitulates KSHV-mediated lymphangiogenesis through the deregulation of HDAC5 activity. Having shown that the inhibition of HDAC5 activity by a class IIa HDAC inhibitor abolishes vIRF3-mediated sprouting formation (Fig. 6D and E), our results convincingly connect class IIa HDACs to the regulation of KSHV-associated pathogenesis. This indicates that the vIRF3-HDAC5 interaction contributes to KSHV-induced lymphangiogenesis, suggesting this interaction as a potential therapeutic target for KS therapy.

## MATERIALS AND METHODS

### Cell culture and cell line construction.

Tetracycline-inducible TRExBCBL-1 V5/vIRF3 cells, which harbor the KSHV genome (35), were cultured in RPMI 1640 medium supplemented with 10% fetal bovine serum (FBS) and 1% penicillin-streptomycin (P/S) (Gibco-BRL). iSLK_BAC16_, iSLK-ΔvIRF3_BAC16_, and iSLK-R-ΔvIRF3_BAC16_ cell lines were maintained in Dulbecco’s modified Eagle’s medium (DMEM) supplemented with 10% FBS and 1% P/S. Human primary dermal lymphatic endothelial cells (LECs) and vascular blood endothelial cells (BECs) were isolated from deidentified neonatal foreskins under an approval of the University of Southern California Institutional Review Board (principal investigator [PI], Y. K. Hong) (36) and cultured in a VascuLife endothelial medium complete kit (Lifeline Cell Technology). Primary LECs and BECs (subjected to fewer than 5 passages) were immortalized with retroviruses (BD Biosciences) encoding human papillomavirus E6/E7 proteins and selected with 200 μg/ml G418. To establish vIRF3-expressing LECs and BECs, E6/E7 immortalized BECs and LECs were infected with V5/vIRF3-expressing lentivirus. At 48 h after transduction, cells were selected using puromycin (0.5 μg/ml) and G418 (200 μg/ml).

### Construction of vIRF3-deficient recombinant KSHV (ΔvIRF3_BAC16_).

The vIRF3-deficient KSHV was generated by changing amino acid 31 of the TCTGAG coding sequence of vIRF3 to TCTTAG, resulting in a stop codon. As previously described, mutagenesis was performed in *Escherichia coli* strain GS1783 by using “scarless” mutagenesis (26). The vIRF3 stop mutant was generated by amplifying a kanamycin resistance (Kan^r^)/I-SecI cassette from the pEP-Kan-S plasmid using the following primers: forward primer 5′-TGACAGGTCAACATGGCGGGACGCAGGCTTACCTGGATTTGCTAGCGTTTATTGTAGGTGCTTTGGaggatgacgacgataagtagg and reverse primer 5′-CAAAGGATATTTATCAGAGTCCAAAGCACCTACAATAAACGCTAGCAAATCCAGGTAAGCCTGCGTaaccaattaaccaattctgattag. Uppercase letters indicate KSHV genomic sequences that were used for homologous recombination, while sequences given in lowercase letters were used to PCR amplify the Kan^r^/I-SceI cassette from the pEP-Kan-S plasmid. Newly generated bacterial artificial chromosome (BAC) DNAs were digested by NheI and AseI restriction enzymes followed by pulsed-field gel electrophoresis performed with direct sequencing to verify that the clones had not suffered genetic rearrangements in the BAC16-vIRF3 stop mutant (ΔvIRF3 BAC16) compared with WT-BAC16.

### Production and infection of recombinant KSHV BAC16.

iSLK_BAC16_, iSLK-ΔvIRF3_BAC16_, and iSLK-R-ΔvIRF3_BAC16_ cells were treated with doxycycline (1 μg/ml) and NaB (1 mM) for 72 h. After virus-containing supernatant was collected, levels of infectious units were determined by analysis of GFP-positive cells in each well that was reinfected with progeny virus by serial dilution. The same amounts of virus were used for infection with LEC-E6/E7 pCDH and LEC-E6/E7 V5/vIRF3 together with Polybrene (8 μM). At 72 h postinfection, the infected cells were evaluated for tube formation or sprouting formation.

### Chemicals and antibodies.

Various chemicals were purchased from the following manufacturers: doxycycline (Doxy), NaB, and PMA from Sigma; class IIa HDAC inhibitor (MC1586) from Tocris Bioscience; and puromycin, hygromycin B, and G418 from Invitrogen. Primary antibodies were purchased from the following manufacturers: HDAC5 and HDAC5 (S498) from Abcam, Inc.; LANA (LNA-1) from Advance; vIRF3 (CM-A807) from Novus; and actin and V5 from Sigma.

### Immunohistochemistry and immunofluorescence.

TMA sections, which were obtained from the AIDS Cancer Specimen Resource (ACSR) of the National Cancer Institute, were deparaffinized, rehydrated, and subjected to antigen retrieval by using sodium citrate buffer (pH 6.0). Primary antibodies consisting of either LANA antibody (1:500) or vIRF3 antibody (CM-A807; 1:750) were used at 4°C overnight, followed by DAB (3,3′-diaminobenzidine) chromogen for color development (Envision; Dako). Images were captured using Aperio digital pathology slide scanners (Leica Biosystems). For immunofluorescence, cells were fixed with 1% paraformaldehyde and permeabilized with 0.2% Triton X-100. Next, cells were incubated with V5 or Flag antibody at 37°C for 1 h followed by incubation with fluorescein isothiocyanate (FITC)-labeled donkey anti-mouse IgG or with rhodamine-labeled donkey anti-rabbit IgG at 37°C for 1 h. To visualize the nucleus, DAPI (4′,6-diamidino-2-phenylindole) was included in the mounting solution.

### Lentiviral shRNA knockdown.

Short hairpin RNA (shRNA) expression constructs were prepared using pLKO.1 lentiviral vector. The shRNA target sequences were as follows: 5′-CAGCAUGACCACCUGACAA-3′ for HDAC5 and 5′-AGCGUGUAGCUAGCAGAGG-3′ for scrambled. Production of lentiviruses and transduction of cells were performed as previously described (37).

### Tube formation and sprouting formation assay.

Tube formation assays using growth factor-reduced matrigel (BD Biosciences) were performed according to the manufacturer’s procedures. Quantification of tube formation was performed using a phase-contrast image and tube formation ACAS image analysis (Ibidi). To perform the sprouting assay, either KSHV-infected LECs or LEC-E6/E7 V5/vIRF3 was placed on 0.5% agarose, embedded into a collagen/Methocel gel (38), and incubated with LEC culture medium for 2 to 5 days.

### RNA purification and RT-qPCR.

Total RNA was isolated using TRIzol (Sigma), and reverse transcription was done using an iScript cDNA synthesis kit (Bio-Rad). Real-time quantitative PCR (qPCR) analysis was performed as described previously (37). The sequences of the primers used for RT-qPCR were as follows: for Prox1, 5′ CCGACGCAAGTTGACGGCTCTCGACTA 3′ (Forward) and 5′ TTGCCTTAAGCATTACCAGGTAATCAT 3′ (Reverse); for LYVE-1, 5′ ATGGCCAGGTGCTTCAGCCTGGTGTTG 3′ (Forward) and 5′ GTGTTGCAGTTTGAGTGTTGAATATGG 3′ (Reverse); for CXCR4, 5′ CACTTCAGATAACTACACCG 3′ (Forward) and 5′ ATCCAGACGCCAACATAGAC 3′ (Reverse); and for neuropilin-1, 5′ CAGGTGATGACTTCCAGCTCA 3′ (Forward) and 5′ CCCAGTGGCAGAAGGTCTTG 3′ (Reverse).

### RNA-Seq data analysis.

Sequencing libraries were prepared from previously purified RNA using an Illumina TruSeq Stranded mRNA Library Prep kit following the manufacturer’s instructions. Sequencing was performed on a NextSeq 500 platform using 1×75bp chemistry. Sequencing reads were aligned to the hg19 genome and transcriptome using TopHat v2.1.0 with parameters '--read-mismatches 2 --read-gap-length 2 --read-edit-dist 2 --max-multihits 10 --library-type fr-firststrand'. Gene-level quantification was performed using htseq-count from the HTSeq framework (v0.6.0). Differential expression analysis was performed using the “DESeq2” R package (version 1.16.1). Results determined for genes with a Benjamini-Hochberg-adjusted *P* value of <0.05 and an absolute fold change value greater than 4 were considered statistically significant. Gene set enrichment analysis (GSEA) was performed using GSEA software package version 2.1.0 with the hallmark gene sets from Molecular Signatures Database v6.1.

### Accession number(s).

RNA-Seq data are available at the Gene Expression Omnibus (GEO) database under accession number GSE102132.
