# Supplementary figures and images for: Deregulation of HDAC5 by Viral Interferon Regulatory Factor 3 Plays an Essential Role in Kaposi's Sarcoma-Associated Herpesvirus-Induced Lymphangiogenesis
Source: mBio. 2018 Jan 16;9(1):e02217-17. doi: 10.1128/mBio.02217-17 (PMC5770555; doi:10.1128/mBio.02217-17)

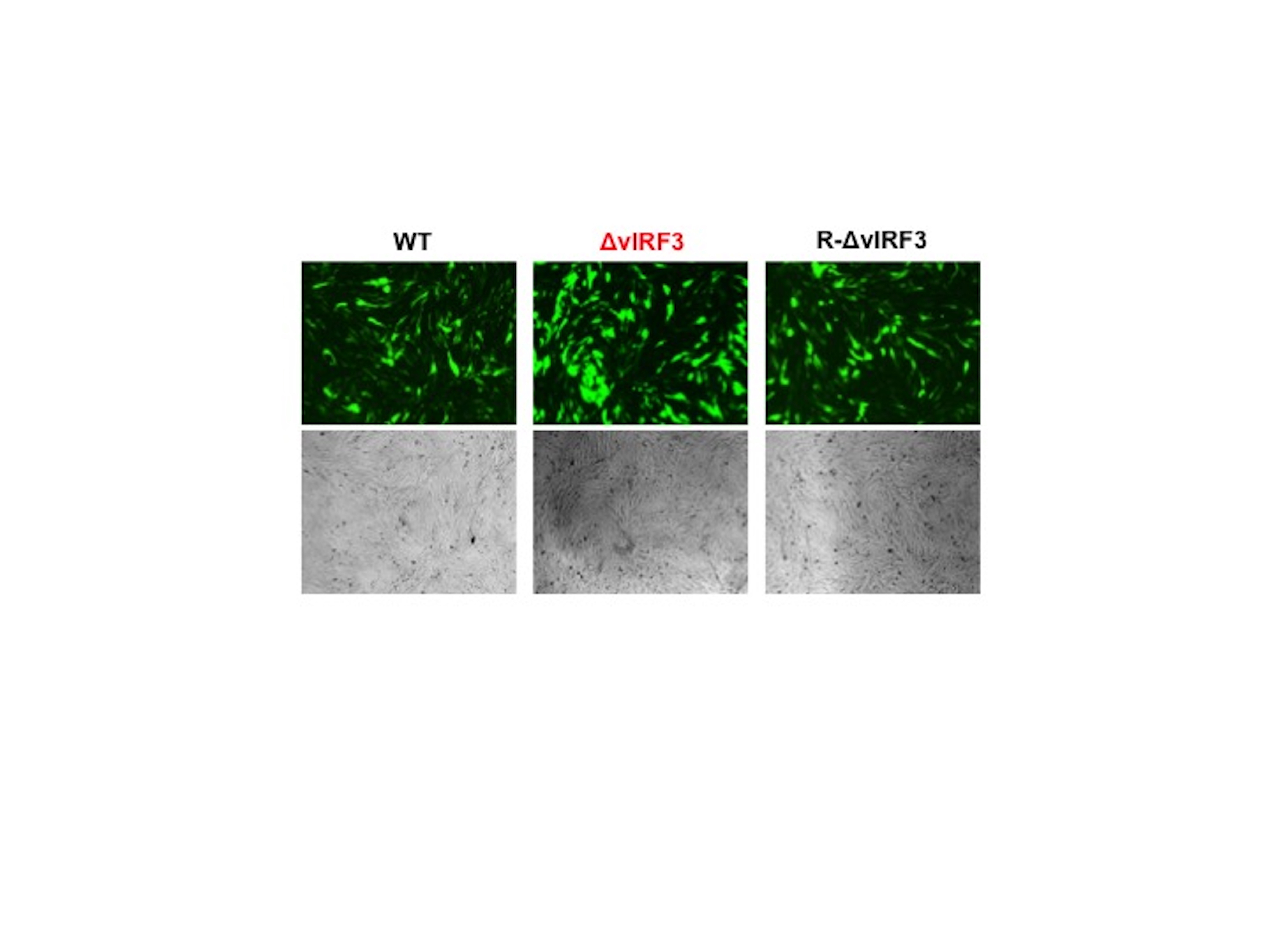

Supplement: FIG S1 [file mbo001183671sf1.jpg]
