# Supplementary material for: Deregulation of HDAC5 by Viral Interferon Regulatory Factor 3 Plays an Essential Role in Kaposi's Sarcoma-Associated Herpesvirus-Induced Lymphangiogenesis
Source: mBio. 2018 Jan 16;9(1):e02217-17. doi: 10.1128/mBio.02217-17 (PMC5770555; doi:10.1128/mBio.02217-17)
Supplement: TABLE S1 [file mbo001183671st1.docx]

**Supplemental Table 1.**

| **LEC** | | |
| --- | --- | --- |
| **EPITHELIAL_MESSENCHYMAL_TRANSITION** | | |
| **NUMBER** | **PROBE** | **RANK METRIC SCORE** |
| 1 | COL11A1 | 0.01239869 |
| 2 | FBLN2 | 0.0259122 |
| 3 | COL1A1 | 0.03608123 |
| 4 | THY1 | 0.04847992 |
| 5 | WNT5A | 0.06032119 |
| 6 | COL3A1 | 0.0732773 |
| 7 | TIMP3 | 0.08288891 |
| 8 | LUM | 0.09082829 |
| 9 | PDGFRB | 0.09653802 |
| 10 | LAMA1 | 0.10057549 |
| 11 | COL7A1 | 0.10628521 |
| 12 | TNC | 0.11310977 |
| 13 | THBS2 | 0.11770467 |
| 14 | PCOLCE | 0.12787369 |
| 15 | COL1A2 | 0.14027238 |
| 16 | SLIT2 | 0.15155624 |
| 17 | EMP3 | 0.15113442 |
| 18 | COL6A3 | 0.15740155 |
| 19 | GAS1 | 0.168128 |
| 20 | FBLN5 | 0.17941186 |
| 21 | IGFBP2 | 0.18679382 |
| 22 | COL6A2 | 0.19807768 |
| 23 | LRP1 | 0.20601706 |
| 24 | FBLN1 | 0.21284162 |
| 25 | IGFBP3 | 0.22078098 |
| 26 | POSTN | 0.2298352 |
| 27 | MATN2 | 0.24056163 |
| 28 | LAMA2 | 0.2512881 |
| 29 | TIMP1 | 0.24640694 |
| 30 | TPM2 | 0.23818134 |
| 31 | LOXL1 | 0.24500588 |
| 32 | PCOLCE2 | 0.2551749 |
| 33 | ECM1 | 0.25252342 |
| 34 | COL5A3 | 0.24597006 |
| 35 | COL12A1 | 0.2522372 |
| 36 | COL5A2 | 0.23620778 |
| 37 | SNAI2 | 0.21571907 |
| 38 | GLIPR1 | 0.2175269 |
| 39 | VEGFA | 0.1897918 |
| 40 | SCG2 | 0.16707343 |
| 41 | VEGFC | 0.17612764 |
| 42 | MEST | 0.18239477 |
| 43 | PLOD2 | 0.1920064 |
| 44 | AREG | 0.19214198 |
| 45 | ID2 | 0.19562204 |
| 46 | MMP2 | 0.20356143 |
| 47 | ACTA2 | 0.20035253 |
| 48 | OXTR | 0.19324175 |
| 49 | PFN2 | 0.20619784 |
| 50 | PVR | 0.20689084 |
| 51 | CXCL1 | 0.20089488 |
| 52 | ITGB3 | 0.18152098 |
| 53 | JUN | 0.18221399 |
| 54 | RGS4 | 0.16897164 |
| 55 | THBS1 | 0.11671036 |
| 56 | NTM | 0.08005665 |
| 57 | ABI3BP | 0.09189792 |
| 58 | PTX3 | 0.10485402 |
| 59 | ITGA2 | 0.10554702 |
| 60 | CXCL6 | 0.11794571 |
| 61 | PMP22 | 0.10080147 |
| 62 | INHBA | 0.11264274 |
| 63 | MSX1 | 0.11166351 |
| 64 | DKK1 | 0.11235651 |
| 65 | TGM2 | 0.12141071 |
| 66 | CAPG | 0.1299075 |
| 67 | PMEPA1 | 0.14007653 |
| 68 | PRRX1 | 0.14188436 |
| 69 | LAMA3 | 0.15539788 |
| 70 | GREM1 | 0.1488445 |
| 71 | COL8A2 | 0.14006147 |
| 72 | IL32 | 0.06717587 |
| 73 | FZD8 | 0.0360963 |
| 74 | MMP1 | 0.00167224 |

| **UV_RESPONSE_DN** | | | | |
| --- | --- | --- | --- | --- |
| **NUMBER** | **PROBE** | | **RANK METRIC SCORE** | |
| 1 | COL11A1 | | 11.1116457 | |
| 2 | COL1A1 | | 10.4579487 | |
| 3 | COL3A1 | | 9.95229626 | |
| 4 | PDGFRB | | 8.61002731 | |
| 5 | COL1A2 | | 7.36856794 | |
| 6 | FBLN5 | | 6.3156414 | |
| 7 | HAS2 | | 6.24459743 | |
| 8 | F3 | | 6.16508055 | |
| 9 | IRS1 | | 5.18381548 | |
| 10 | ARHGEF9 | | 5.13861036 | |
| 11 | GCNT1 | | 5.09198523 | |
| 12 | ERBB2 | | 3.89382815 | |
| 13 | BMPR1A | | 3.70014 | |
| 14 | COL5A2 | | 3.31168175 | |
| 15 | SNAI2 | | 2.9819665 | |
| 16 | PLCB4 | | 2.97625113 | |
| 17 | SLC22A18 | | 2.93482947 | |
| 18 | SDC2 | | 2.92096257 | |
| 19 | KCNMA1 | | 2.91943812 | |
| 20 | LPAR1 | | 2.8596139 | |
| 21 | MAGI2 | | 2.76451063 | |
| 22 | MGMT | | 2.25124979 | |
| 23 | FZD2 | | 2.21483779 | |
| 24 | VLDLR | | -2.00020623 | |
| 25 | PIK3CD | | -2.06660342 | |
| 26 | ITGB3 | | -2.22828579 | |
| 27 | MGLL | | -2.24361539 | |
| 28 | RGS4 | | -2.41493726 | |
| 29 | GRK5 | | -2.6662643 | |
| 30 | SYNJ2 | | -2.77141929 | |
| 31 | PRKAR2B | | -3.05624628 | |
| 32 | KALRN | | -3.22546244 | |
| 33 | PMP22 | | -3.29123569 | |
| 34 | NFIB | | -3.2927413 | |
| 35 | EFEMP1 | | -5.07614899 | |
| 36 | IGFBP5 | | -5.59313107 | |
| 37 | CELF2 | | -6.58367062 | |
|  | |  | |  |
|  | |  | |  |
| **APICAL_SURFACE** | | | | |
| **NUMBER** | **PROBE** | | **RANK MERTIC SCORE** | |
| 1 | THY1 | | 10.3006954 | |
| 2 | RTN4RL1 | | 9.47720432 | |
| 3 | AFAP1L2 | | 8.81643486 | |
| 4 | GAS1 | | 6.42032194 | |
| 5 | LYN | | -2.07381392 | |
| 6 | SULF2 | | -6.50485563 | |

| **BEC** | | |
| --- | --- | --- |
| **INTERFERON_GAMMA_RESPONSE** | | |
| **NUMBER** | **PROBE** | **RANK MERTIC SCORE** |
| 1 | SELP | -2.217780352 |
| 2 | IFI44L | -2.321118116 |
| 3 | IFIT1 | -2.38645339 |
| 4 | MX1 | -2.41473794 |
| 5 | CMPK2 | -2.631327868 |
| 6 | IRF8 | -2.802182436 |
| 7 | BST2 | -2.931330442 |
| 8 | RSAD2 | -2.98090601 |
| 9 | MX2 | -3.085595369 |
|  |  |  |
|  |  |  |
| **INTERFERON_ALPHA_RESPONSE** | | |
| **NUMBER** | **PROBE** | **RANK MERTIC SCORE** |
| 1 | IFI44L | -2.3211181 |
| 2 | MX1 | -2.4147379 |
| 3 | CMPK2 | -2.6313279 |
| 4 | BST2 | -2.9313304 |
| 5 | RSAD2 | -2.980906 |
| 6 | IFITM1 | -3.8437636 |
|  |  |  |
|  |  |  |
| **KRAS_SIGNALING_DN** | | |
| **NUMBER** | **PROBE** | **RANK MERTIC SCORE** |
| 1 | FAM46C | -2.227438 |
| 2 | IFI44L | -2.3211181 |
| 3 | MX1 | -2.4147379 |
| 4 | PKP1 | -2.8508208 |
| 5 | RSAD2 | -2.980906 |
|  |  |  |
|  |  |  |
| **INFLAMMATORY_RESPONSE** | | |
| **NUMBER** | **PROBE** | **RANK MERTIC SCORE** |
| 1 | IL1B | -2.195126772 |
| 2 | LIF | -2.344139576 |
| 3 | TNFSF15 | -2.476103544 |
| 4 | BST2 | -2.931330442 |
| 5 | IFITM1 | -3.84376359 |

| **MYOGENESIS** | | |
| --- | --- | --- |
| **NUMBER** | **PROBE** | **RANK MERTIC SCORE** |
| 1 | COL1A1 | 10.4579487 |
| 2 | COL3A1 | 9.95229626 |
| 3 | BDKRB2 | 9.05387115 |
| 4 | DMD | 7.79505444 |
| 5 | DES | 7.50638151 |
| 6 | ITGA7 | 7.00258589 |
| 7 | HSPB2 | 6.84319544 |
| 8 | COL6A3 | 6.55758524 |
| 9 | SOD3 | 6.33536148 |
| 10 | COL6A2 | 6.07111406 |
| 11 | IGFBP3 | 5.51557922 |
| 12 | LAMA2 | 5.14823294 |
| 13 | TPM2 | 4.36661673 |
| 14 | SPEG | 4.17812824 |
| 15 | FST | 3.96093535 |
| 16 | APOD | 3.40924811 |
| 17 | CTF1 | 3.01568484 |
| 18 | MRAS | 2.95562148 |
| 19 | CKB | 2.74851203 |
| 20 | PPP1R3C | 2.4080193 |
| 21 | ST5 | 2.35597086 |
| 22 | BIN1 | 2.22029853 |
| 23 | PKIA | -2.10207725 |
| 24 | CLU | -2.21229792 |
| 25 | FABP3 | -2.28816152 |
| 26 | SORBS1 | -2.49001026 |
| 27 | NOTCH1 | -3.04975367 |
| 28 | DAPK2 | -3.05067897 |
| 29 | STC2 | -3.05489302 |
| 30 | GPX3 | -3.51645017 |
| 31 | CDH13 | -4.60077429 |
| 32 | COX7A1 | -4.83446884 |
| 33 | HSPB8 | -5.40893269 |
| 34 | VIPR1 | -5.61623383 |
